# LHX2 Is a Potential Biomarker and Associated with Immune Infiltration in Breast Cancer

**DOI:** 10.3390/cancers15102773

**Published:** 2023-05-16

**Authors:** Ziwei Zhang, Minghao Gu, Gao He, Xiafei Yu, Junzhe Yang, Xian Wu, Xiaoqiang Zhang, Kaining Lu, Fangze Qian, Xiaoyue Shi, Jialu Xu, Minyu Zhuang, Xiaoan Liu, Yanhui Zhu

**Affiliations:** Department of Breast Surgery, The First Affiliated Hospital of Nanjing Medical University, 300 Guangzhou Road, Nanjing 210029, China

**Keywords:** LHX2, breast cancer, immune infiltration, prognosis, pathway

## Abstract

**Simple Summary:**

Breast cancer is one of the most common malignant tumors in women, and metastatic breast cancer usually has a poor prognosis. Hence, it is urgent to identify new biomarkers to promote precise medication in breast cancer. LHX2 has been reported to promote the proliferation of numerous solid tumors, but its role in breast cancer has not been elucidated. Our study explored the prognostic value and the immune cell infiltration of breast cancer related to LHX2. We found that LHX2 can serve as an independent prognostic factor in breast cancer. In vitro and in vivo experiments confirmed that LHX2 promoted cell proliferation, migration ability, and invasive ability but inhibited apoptosis in breast cancer. Immunohistochemistry and immunofluorescence experiments were conducted to investigate LHX2-related immune infiltration in breast cancer tissues. A Western blot assay proved that LHX2 activated the PI3K/AKT/mTOR pathway and the apoptosis pathway.

**Abstract:**

Worldwide, breast cancer is the most common malignancy. LHX2, a member of the LIM homeobox gene family and a transcription factor, plays a crucial role in numerous tumors, but the function of LHX2 in breast cancer progression remains unknown. In this study, we show that LHX2 is upregulated in breast cancer tissues and positively correlated with breast cancer progression. Meanwhile, the clinical characteristics of breast cancer and LHX2 expression showed a strong correlation. GSEA showed that a high LHX2 expression may activate the T-cell activation pathway, PI3K/AKT/mTOR signaling pathway, and apoptosis pathway. Moreover, ssGSEA showed that Th1 cells and Th2 cells had a positive correlation with LHX2 expression in breast cancer. Experiments showed that LHX2 promotes the proliferation, colony formation, migration, and invasion of breast cancer cells. Immunohistochemistry and immunofluorescence assays helped to analyze LHX2-associated immune infiltration in breast cancer. A Western blot assay proved that LHX2 activated the PI3K/AKT/mTOR pathway and the apoptosis pathway. A TUNEL assay confirmed that LHX2 inhibited apoptosis. Taken together, LHX2 plays a vital role in breast cancer’s progression and prognosis and could be an immune infiltration biomarker for breast cancer, and LHX2 activates the PI3K/AKT/mTOR pathway and apoptosis pathway in breast cancer.

## 1. Introduction

Breast cancer (BC) is the most common type of malignant tumor in women, with high heterogeneity and a high tendency for metastasis [1]. Despite the significant progression in BC therapy, the prognoses for some patients with breast cancer are still poor [2]. Metastasis is the principal cause of BC’s high mortality rate [3]. Many patients miss timely treatment because of the lack of an accurate diagnosis. Evidently, there is an urgent need to identify new biomarkers to promote precise medication in BC.

LIM-homeobox gene 2 (LHX2), a member of the LIM gene family, has been identified due to its homeobox domain and cysteine-rich LIM domain. LHX2 functions as a nuclear transcription co-factor and is reported to be involved in many important physiological processes, such as cell division, cell proliferation, specific cell-type differentiation, and cerebral cortex development [4]. In addition to its specific physiological functions, LHX2 is also implicated in the occurrence and development of esophageal squamous cell carcinoma and hepatocellular carcinoma [5,6]. However, its potential function and immunology mechanism in breast cancer remain unclear.

In this study, we first analyzed the expression levels of LHX2 in many malignancies by conducting a pan-cancer analysis. Then, we explored its prognostic value and association with the clinicopathological features of breast invasive carcinoma (BRCA). Given its great prognostic value and strong clinicopathological correlation, we further examined the functions of LHX2 in BRCA by utilizing a gene oncology (GO) enrichment analysis, Kyoto encyclopedia of genes and genomes (KEGG) analysis, and gene set enrichment analysis (GSEA). The results show that LHX2 mainly assisted immune-related pathways and tumor-related pathways. Then, we further analyzed the immune infiltration of BRCA related to LHX2. The PI3K/AKT/mTOR pathway and apoptosis pathway were also enriched in high-LHX2-expression groups. We later investigated the complex relations among the PI3K/AKT/mTOR pathway, apoptosis pathway, and immune infiltration of BRCA. Finally, we confirmed that LHX2 promoted the cell proliferation, migration ability, and invasion ability of breast cancer through in vitro and in vivo experiments. Furthermore, immunohistochemistry and immunofluorescence experiments verified that LHX2 promoted the expressions of infiltrating T cells and CD4 + T cells in breast cancer, and a Western blot assay demonstrated that LHX2 activates the PI3K/AKT/mTOR pathway and apoptosis pathway. Using a TUNEL assay, LHX2 was confirmed to inhibit apoptosis.

## 2. Materials and Methods

### 2.1. Data Resources

All of the mRNA expression data were downloaded from the TCGA database (https://portal.gdc.cancer.govz accessed on 8 August 2022). The data set we used was showed in Table S2. The human protein atlas (https://www.proteinatlas.org accessed on 8 August 2022) was employed to examine the subcellular localization of LHX2 in BRAC.

### 2.2. Differential Expression of *LHX2*

We performed pan-cancer analyses to compare the expressions of LHX2 in tumor and normal tissues in the TCGA database, using the Wilcoxon rank sum test. The same method was employed to detect the differential expression of LHX2 in unpaired samples of BRCA. Paired samples were analyzed using the Wilcoxon signed rank test, and boxplots and scatterplots were produced using the ggplot2 package (3.3.3). We estimated the diagnostic performance of LHX2 using receiver operating characteristic (ROC) curves with the pROC package (1.17.0.1).

### 2.3. Prognostic Analyses of Different Malignancies

We conducted a log-rank test to examine the correlation between LHX2 expression and the overall survival and disease-specific survival of patients. The datasets were sorted by LHX2 expression level from low to high. The expression level of 0–50% was considered the low-LHX2-expression group, and the expression level of 50% to 100% was considered the high-LHX2-expression group. Kaplan–Meier curves were calculated to show the difference between the LHX2-low and LHX2-high groups in adrenocortical carcinoma (ACC), BRCA, esophageal adenocarcinoma (ESAD), low-grade glioma and glioblastoma (GBMLGG), kidney renal clear cell carcinoma (KIRC), and mesothelioma (MESO) by employing survival R packages (3.2-10). Then, we used the Cox regression module to evaluate the influence of LHX2 and clinical variables on the prognosis of BRCA. Based on univariate and multivariate analyses, we constructed a nomogram with the RMS package (6.2-0) and survival package (3.2-10) to quantify the prognosis of BRCA. The survminer R package (0.4.9) and ggplot2 R package (3.3.3) greatly assisted in creating the plots. The sample size of each subgroup is shown in Supplementary Table S1.

### 2.4. Analyses of Clinicopathological Features of BRCA

The Wilcoxon rank sum test was utilized to calculate the correlation between multiple clinicopathological features of BRCA and the expression of LHX2. The ggplot2 R package (3.3.3) was used for data visualization. The sample size of each subgroup is demonstrated in Supplementary Table S1.

### 2.5. Detection of Differential Expressed Genes (DEGs) and Functional Enrichment Analyses

DEGs between high-LHX2-expression and low-LHX2-expression BRCA groups were identified using the unpaired Student’s *t*-test within the DESeq2 package. Genes with an adjusted *p* value of less than 0.05 and a log fold change larger than 1.5 were considered to have a statistically significant differential expression. All the DEGs are presented in volcano plots. We performed a GO analysis to make annotations for these DEGs. GSEA was used to enrich the corresponding pathways with LHX2. A false discovery rate less of than 0.25 and an adjusted *p* value of less than 0.05 were considered to be statistically significant. Both the GO analysis and GSEA analysis were performed using the clusterProfiler R package (3.14.3).

### 2.6. Immune Cell Infiltration

We performed a single-sample gene set enrichment analysis (ssGSEA) using the GSVA R package to ascertain the correlation between the expression of LHX2 and the infiltration levels of 24 kinds of immune cells in BRCA. The differential enrichments of immune cells between the high-LHX2-expression and low-LHX2-expression groups were detected using the Wilcoxon rank sum test. Spearman’s correlation revealed the relationship between the enrichment of immune cells and the expression of LHX2.

### 2.7. Cell Culture

Breast cell lines BT-549 and SKBR-3 and murine cell line 4T1 were purchased from the Shanghai Cell Bank of the Chinese Academy of Sciences. All cells were cultured in DMEM with 10% fetal bovine serum in a 37 °C constant temperature incubator containing 5% CO_2_.

### 2.8. Real-Time Quantitative PCR

The RNA expression of LHX2 in the breast cancer cell lines was detected using real-time quantitative PCR. We extracted total RNA using the TRIzol reagent (Invitrogen, Carlsbad, CA, USA) and synthesized cDNA using HiScript II Reverse Transcriptase (Vazyme, Nanjing, China). The real-time quantitative PCR was performed with Hieff^®^ qPCR SYBR Green Master Mix (Yishen, Shanghai, China). The relative mRNA expression level was standardized using GAPDH and analyzed by using the 2^−ΔΔCt^ method. All samples were detected in triplicate. The primer sequences used in the real-time quantitative PCR are shown in Supplementary Table S3.

### 2.9. Transfection

The siRNAs and pcDNA 3.1 plasmids of LHX2 were purchased from Tsingke Biotechnology Co., Ltd. (Beijing, China). The siRNAs were transfected with the lipofectamine 2000 reagent (Invitrogen, Carlsbad, CA, USA), and the pcDNA plasmids were transfected with linear polyethylenimine (PEI) MW40000 (Yeasen Biotechnology Co., Ltd. (Shanghai, China)) according to the manufacturers’ protocols. The sequences of two small interfering RNAs (siRNAs) designed to knock down LHX2 are shown in Supplementary Table S3.

### 2.10. CCK-8 Assay

Cell viability was analyzed using a Cell Counting Kit-8 (CCK8, Beyotime, Shanghai, China) according to the manufacturer’s protocols. The cells were seeded and cultured at a density of 2 × 10^3^/well in 200 μL of the medium into 96-well microplates (Corning, New York, NY, USA) at 24 h after transfection. The absorbance was analyzed at 450 nm using a microplate reader (Bio-Rad, Hercules, CA, USA) at the times of 0, 24, 48, 72, and 96 h. Five duplicates were detected at each time point.

### 2.11. 5-Ethynyl-2′-Deoxyuridine (EdU) Assay

The effect of LHX2 on the proliferation of SKBR-3 and BT-549 cells was evaluated via a 5-Ethynyl-2′-deoxyuridine (EdU) incorporation assay, using an EdU assay kit (Ribobio, Guangzhou, China) according to the manufacturer’s protocol. BT-549 cells were cultured in 96-well plates at 5 × 10^4^ cells/well, and SKBR-3 cells were cultured at 8 × 10^4^ cells/well 24 h after transfection. Three re-wells were laid each time, and all experiments were repeated three times. Finally, the nuclei of the cells were dyed with 100 μL of Hoechst 33,342 (5 μg/mL) for 20 min and visualized with fluorescent microscopy (IX71; Olympus, Tokyo, Japan).

### 2.12. Colony Formation Assay

Next, 2 mL of cell suspension containing 2000 cells was inoculated into 6-well plates for a continuous culture until visible clones appeared. Then, the cells were fixed with 4% paraformaldehyde and stained with a 0.1% crystal violet solution for 15 min. After washing twice with PBS, the plates were photographed using a digital camera. Positive colony formation, defined as colonies with more than 50 cells, was determined via manual counting.

### 2.13. Transwell Assay

To assess invasion ability, the upper chamber of each filter was covered with 10 μg of Matrigel (BD), which was evenly spread and placed in a 37 °C incubator for 2 h. The serum-free cell suspension was then added to the filter, and the lower chamber was filled with 10% FBS. After 24 h of incubation at 37 °C, the non-invasive cells were swabbed from the upper chamber. Then, the cells on the lower side of the filter were fixed with 4% paraformaldehyde for 30 min and dyed with a 0.03% crystal violet solution for 10 min. Three fields of adherent cells in each well were randomly photographed and counted. The same experiment was conducted to assess migration ability but without the use of filters pre-coated with Matrigel.

### 2.14. Wound Healing Assay

After transfection, the SKBR-3 and BT-549 cells were seeded in 6-well plates (1 × 10^6^ cells/well) 24 h before the experiment. We drew three lines on the back of the six-well plate as reference lines. The next day, we scratched a straight line in the cells in the middle of the six-well plate with a 100 μL pipette tip, and the scraped cells were washed off with PBS. The wounds of each group were recorded at 0 h and 6 h according to the position of the reference line marker. This was repeated three times for each set. The wound healing area was quantified using Image J 2.3.0.

### 2.15. Flow Cytometry

SKBR-3 (1.5 × 10^5^ cells/well) and BT549 (1 × 10^5^ cells/ well) cells were seeded in 6-well plates. After 48 h of treatment, the cells were labeled using an Annexin V-FITC/PI Apoptosis Detection Kit (Beyotime, Shanghai, China), and cell staining was performed according to the manufacturer’s protocol. The cells were evaluated using flow cytometry (FACS Calibur, BD Biosciences, San Jose, CA, USA).

### 2.16. In Vivo Assay

SKBR-3, BT-549 cells, and 4T1 cells were infected with the obtained lentiviruses and stably expressed sh-NC and sh-LHX2 after puromycin filtration. All animal experiments were approved by the Ethics Committee of Nanjing Medical University. All animals were purchased from Beijing Vital River Laboratory Animal Technology Co., Ltd. (Beijing, China). SKBR-3 and BT-549 cells (5 × 10^6^ cells resuspended in 100 μL of PBS) were inoculated subcutaneously in the axilla of BALB/c nude mice (4-week-old, female), and 4T1 cells (5 × 10^6^ cells resuspended in 100 μL of PBS) were inoculated subcutaneously in the axilla of C57BL/6 mice (5-week-old, female). Each group had five replicates. We incubated the si-NC group in the left axilla, and si-LHX2 in the right. We used calipers to measure tumor diameter every 3 days, and we calculated the tumor volume using the following formula: (shortest diameter)/2 × (longest diameter)/2. Three weeks after inoculation, we weighed the tumors.

### 2.17. Western Blotting Assay

An equal amount of protein was subjected to 10% SDS-PAGE and then transferred to a 0.45 μm pore size PVDF membrane (Millipore, Billerica, MA, USA). After blocking with 5% non-fat milk, the membrane was incubated with primary antibodies at 4 °C overnight and with secondary antibodies at room temperature for 1 h. Bound antibodies were detected using an ECL Plus Western blotting substrate (ThermoFisher, Waltham, MA, USA) and using the enhanced chemiluminescence detection system (ThermoFisher, Waltham, MA, USA). Band densities were quantified using ImageJ software. The relative amount of protein was determined by normalizing the densitometry value of interest to that of the loading control. All the antibodies used in the Western blotting assay are shown in Supplementary Table S3.

### 2.18. Immunohistochemistry (IHC)

Paraffin-embedded sections were first deparaffinized with xylene and then hydrated with gradient ethanol, followed by the removal of endogenous catalase. After antigen retrieval and blocking, primary antibodies were incubated overnight at 4 °C (antibodies are shown in Supplementary Table S3), and an HRP-polymer mouse/rabbit kit (AiFang, AFIHC001, Changsha, China) was used for 1 h at room temperature. IHC staining was conducted using a DAB chromogenic kit (AiFang, AFIHC004, China) and a hematoxylin staining solution (AiFang, AFIHC005, China). The IHC results were quantified using Image J.

### 2.19. Immunofluorescence

The paraffin sections were deparaffinized and hydrated, antigen-repaired, and sequentially blocked with hydrogen peroxide and serum. The sections were then incubated with the CD3 primary antibody (4 °C overnight), and the secondary antibody (488 Goat Anti-Rabbit IgG, green) was incubated the next day (1 h at room temperature). Then, the sections were microwaved, and they were blocked with serum. The CD4 antibody and the corresponding secondary antibody (HRP Goat-Anti-Rabbit IgG) were incubated into the sections, followed by the addition of 50 μL TYR-570 fluorescent dye (red) (AiFang, China). All the antibodies are shown in Supplementary Table S3. Finally, the nuclei were counterstained with DAPI (C1002, Beyotime, China), and the slides were sealed after self-light fluorescence quenching. The images were taken with a positive fluorescence microscope (NIKON ECLIPSE C1, Tokyo, Japan), and the resulting images were quantified using Image J.

### 2.20. TdT-Mediated dUTP Nick-End Labeling (TUNEL) Assay

The paraffin sections were deparaffinized to water and then subjected to proteinase K (ST533, Beyotime, China) repair, followed by membrane rupture with a membrane-breaking fluid (G1204, Servicebio, Wuhan, China) and the addition of a TUNEL reaction solution (C1088, Beyotime, China). Finally, the sections were sealed after DAPI (C1002, Beyotime, China) counterstaining. Pictures were taken with a positive fluorescence microscope (NIKON ECLIPSE C1, Japan), and they were quantified using Image J.

### 2.21. Statistical Analysis

Statistical analyses were performed using R project (3.6.3) or GraphPad Prism 9.0 (GraphPad Software, San, Diego, CA, USA). All experimental results were analyzed using GraphPad Prism 9.0 (GraphPad Software, USA). The significance of the differences between the experimental groups was estimated by using Student’s *t*-test or the Wilcoxon test, where appropriate.

## 3. Results

### 3.1. LHX2 Is Highly Expressed in BRCA

**Figure 1:**
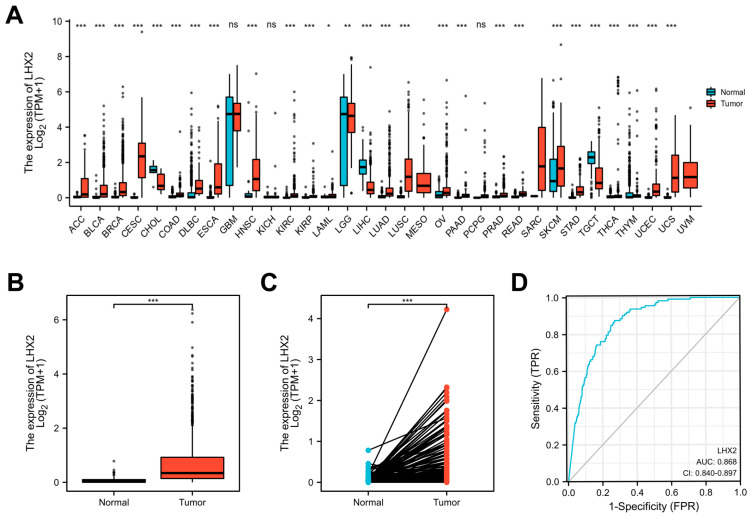
Differential expression levels of LHX2 in different malignancies. (**A**) The expression of LHX2 in various cancers compared with in normal tissues in TCGA database. (**B**,**C**) Differential expression level of LHX2 in BRCA. (**D**) An ROC curve to test the value of LHX2 in identifying BRCA tissues. * *p* < 0.05, ** *p* < 0.01, *** *p* < 0.001.

With the Wilcoxon rank sum test, we first utilized pan-cancer analyses to demonstrate LHX2 mRNA expression in different tumors. The mRNA expression of LHX2 was greatly increased in BRCA, ACC, bladder urothelial carcinoma (BLCA), cervical squamous cell carcinoma and adenocarcinoma (CESC), and most other tumors, despite the opposite results appearing in a few types of tumors (Figure 1A). Moreover, we compared the expression of LHX2 in 1109 breast cancer tissue samples and 113 para-cancerous samples in the TCGA BRCA dataset, and we found that LHX2 expression was upregulated in breast cancer tissues compared to normal tissues (Figure 1B). We obtained the same result when comparing 113 paired samples of BRCA (Figure 1C). Moreover, an ROC curve was calculated to explore the diagnostic ability of LHX2. The AUC (area under the curve) was 0.868, suggesting that LHX2 could be a diagnostic molecule for BRCA (Figure 1D).

### 3.2. Prognostic Value of LHX2 in BRCA

**Figure 2:**
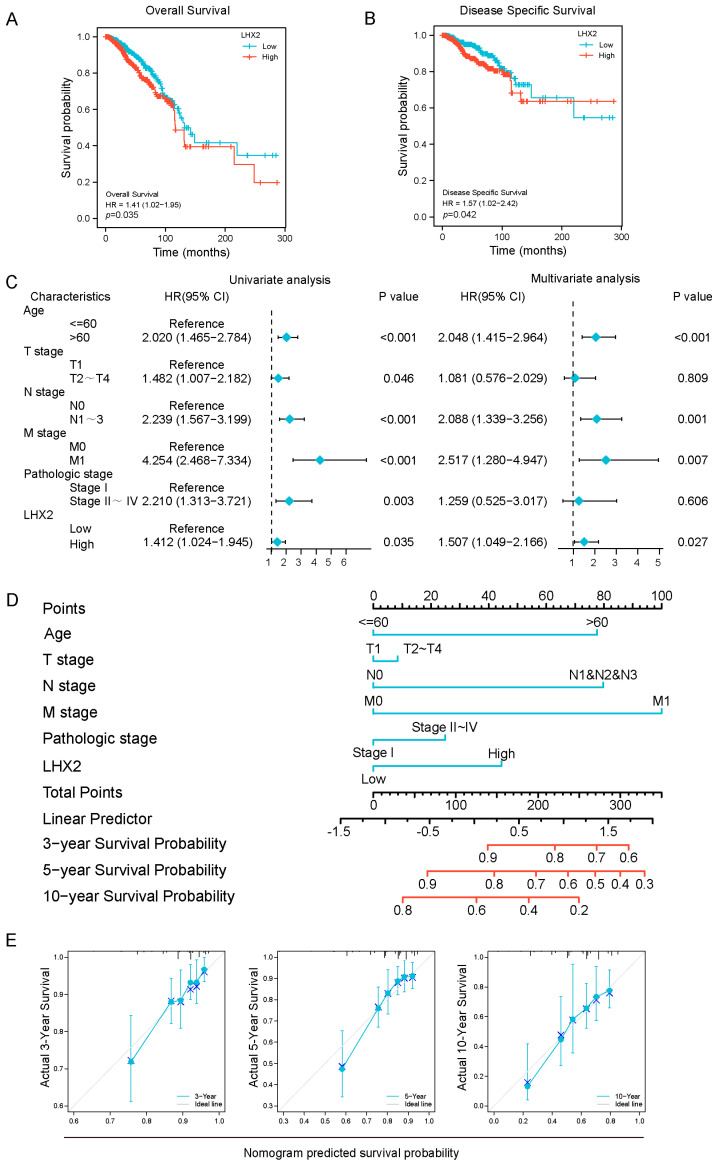
Prognostic analysis of LHX2 in BRCA. (**A**,**B**) Kaplan–Meier overall survival (OS) and disease-specific survival (DSS) for samples in high- and low-LHX2 groups in BRCA in TCGA database. (**C**) LHX2 was found to be an independent prognostic factor using univariate and multivariate Cox regression analyses. (**D**) A nomogram based on LHX2, age, T stage, N stage, M stage, and pathological stage. (**E**) Calibration plots of the nomogram for predicting the probability of OS at 3, 5, and 10 years.

To explore the prognostic value of LHX2, we analyzed the overall survival (OS) and disease-specific survival (DSS) related to the expression of *LHX2* in malignancies. We found that a high expression of LHX2 in ACC, ESAD, GBMLGG, KIRC, and MESO indicated poor OS (*p* < 0.05). Similarly, DSS was significantly higher in the LHX2-low groups (*p* < 0.05) (Figure S1). It was notable that LHX2 was a hazard in patients with BRCA, where a higher *LHX2* expression indicated poor OS (*p* = 0.035; Figure 2A) and DSS (*p* = 0.042; Figure 2B).

Then, we conducted univariate and multivariate Cox regression analyses to further determine the prognostic significance of LHX2 in BRCA. In the univariate analyses, LHX2 was significantly associated with OS (HR: 1.412, 95% CI: 1.024–1.945, *p* = 0.035; Figure 2C). The multivariate Cox regression analyses illustrated that LHX2 had an independent capacity for the prognosis prediction of BRCA (HR: 1.507, 95% CI: 1.049–2.166, *p* = 0.027; Figure 2C).

To quantitatively predict the prognosis of BRCA, we constructed a nomogram with LHX2 and the clinical prognostic factors from the univariate and multivariate analyses; the C-index was 0.699 (0.672–0.726). The probability of survival of patients with BRCA at 3, 5, and 10 years was also determined (Figure 2D). This nomogram’s ability to predict 3 years’, 5 years’, and 10 years’ survival is indicated by calibration plots (Figure 2E).

### 3.3. Association between LHX2 Expression and Clinicopathological Features

**Figure 3:**
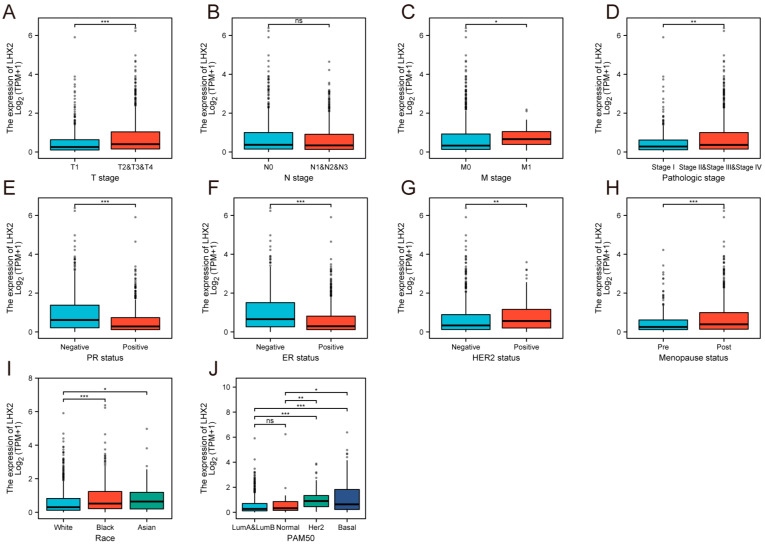
Correlation between the expression of LHX2 and clinicopathological characteristics of BRCA, including (**A**) T stage, (**B**) N stage, (**C**) M stage, (**D**) pathological stage, (**E**) PR status, (**F**) ER status, (**G**) HER2 status, (**H**) menopause status, (**I**) race, and (**J**) PAM50 in TCGA database. * *p* < 0.05, ** *p* < 0.01, *** *p* < 0.001.

In total, 1222 samples with LHX2 expression were collected from TCGA and analyzed using the Wilcoxon rank sum test. The expression of LHX2 was higher in larger tumors with distant metastasis than in small tumors with no remote lesions (Figure 3A,C). LHX2 had a lower expression in pathological stage Ⅰ than in other stages (Figure 3D). However, LHX2 expression showed little difference in the lymphatic metastasis of BRCA (Figure 3B). In addition, LHX2 expression was upgraded in HER-2 (+) samples while being downgraded in PR (+) and ER (+) samples (Figure 3E–G). According to the PAM50 grading system, we consistently found that LHX2 was highly expressed in Her-2 (+) and basal-like samples (Figure 3J). HER-2 positive breast cancer and basal-like breast cancer are more likely to harbor TILs in the TME, and they receive more clinical benefits by using immunotherapy than luminal breast cancer [7]. Interestingly, samples from post-menopausal women seemed to have higher levels of LHX2 expression than those from young women (Figure 3H), which indicates a hypothetical connection between LHX2 expression and the level of estrogen and progesterone.

### 3.4. Subcellular Location of LHX2 and Functional Enrichment Analyses of LHX2-Related Genes in BRCA

**Figure 4:**
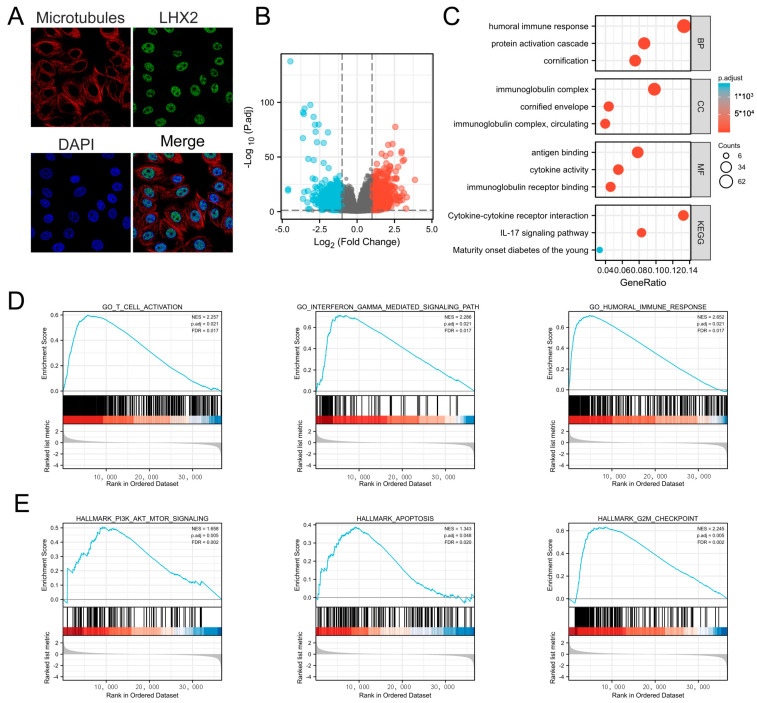
Subcellular location and functional enrichment analysis of LHX2-related DEGs in BRCA. (**A**) Subcellular location of LHX2 in MCF-7 cells. (**B**) Volcano plots of the LHX2-related differential expressed genes (DEGs). (**C**) GO annotations and pathways enriched by GO and KEGG analyses of LHX2-related genes in BRCA. (**D**,**E**) Significantly enriched pathways in high-LHX2-expression phenotype from GSEA.

To further clarify the function of *LHX2*, we confirmed the subcellular location of *LHX2*. It is mainly localized to the cell nucleus according to the human protein atlas (https://www.proteinatlas.org/ENSG00000106689-LHX2/subcellular#human accessed on 23 April 2023) (Figure 4A) and usually acts as a transcription factor in the cell nucleus [8]. Then, we calculated the DEGs between the *LHX2*-high and *LHX2*-low groups in BRCA (adjusted *p*-value < 0.05, |Log2-fold change| > 1.5) (Figure 4B). Subsequently, we conducted GO and KEGG enrichment analyses to explore the functional enrichment information of *LHX2*-related genes. In terms of biological processes, *LHX2*-related genes are involved in the humoral immune response, the protein activation cascade, and cornification. With regard to cell components, *LHX2*-related genes are gathered in the immunoglobulin complex, cornified envelope, and immunoglobulin complex, circulating. Regarding molecular functions, these genes are enriched in antigen binding, cytokine activity, and immunoglobulin receptor binding. The KEGG analysis showed that LHX2-related genes were enriched in the cytokine–cytokine receptor interaction, the IL-17 signaling pathway, and maturity-onset diabetes of the young (Figure 4C). Finally, GSEA illustrated significantly enriched pathways in the high-*LHX2*-expression phenotype, including the T-cell activation pathway, interferon-gamma-mediated signaling pathway, humoral immune response pathway, PI3K/AKT/mTOR signaling pathway, apoptosis pathway, and G2M checkpoint pathway (Figure 4D,E).

### 3.5. Immune Infiltration Correlated with LHX2 Expression in BRCA

**Figure 5:**
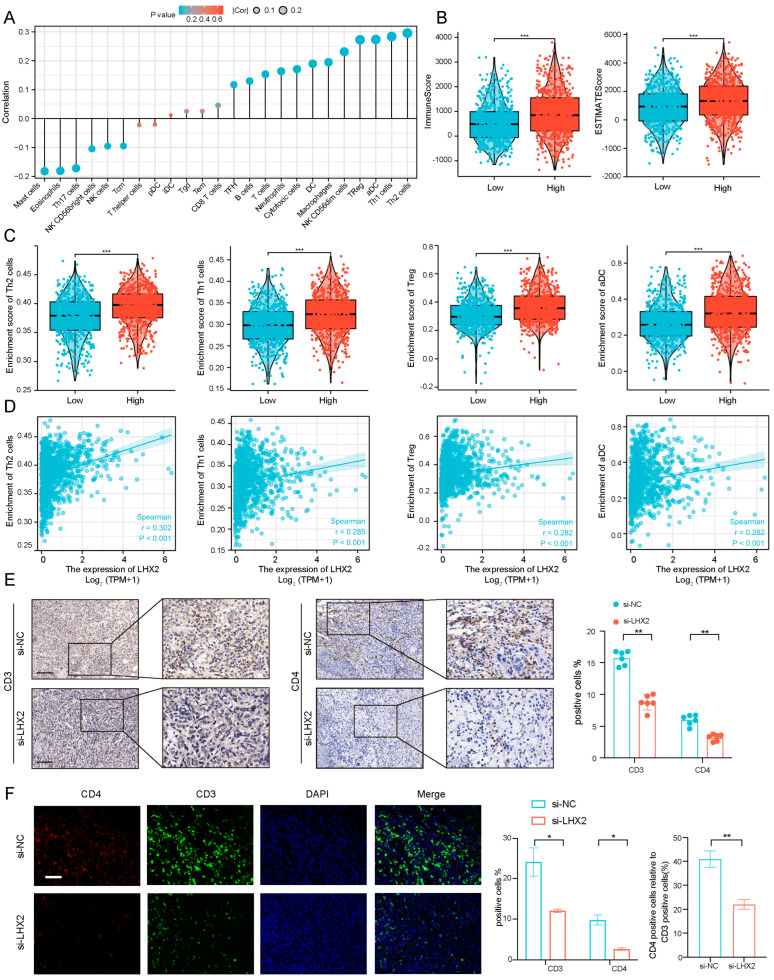
The expression level of LHX2 was correlated with the immune infiltration of BRCA. (**A**) Correlation between 24 immune cells and the expression of LHX2 in BRCA. (**B**) The immune score and ESTIMATE score in low-LHX2-expression and high-LHX2-expression groups in BRCA. *** *p* < 0.001. (**C**) The plots show the differential infiltration levels of Th2 cells, Th1 cells, Treg cells, and activated DC cells between LHX2-low and -high groups. *** *p* < 0.001. (**D**) Correlation diagrams illustrate the positive correlation between enrichment of Th2 cells, Th1 cells, Treg cells, and activated DC cells and the expression of LHX2. (**E**) Immunohistochemistry analyses of CD3 and CD4 protein expressions in si-NC and si-LHX2 groups. Data represent mean ± SEM, ** *p* < 0.01, scale bar 50 μM. (**F**) Double immunofluorescence labeling of CD3 and CD4 protein expressions in si-NC and si-LHX2 groups. Data represent mean ± SEM, * *p* < 0.05, ** *p* < 0.01, scale bar 20 μM.

According to the function enrichment analyses, the function of LHX2 may center on the immunity of BRCA. Immune infiltration is quite complex in the tumor microenvironment (TME). Tumor-infiltrating lymphocytes (TILs) are a vital part of the TME, and they have received great attention due to the clinical success of immune checkpoint blockades. The absence of TILs results in a poor immune checkpoint blockade response [9,10,11]. Hence, we further explored the immune infiltration of BRCA corresponding with LHX2 expression. In our study, we demonstrated the correlation between LHX2 expression in BRCA and all kinds of immune cells (Figure 5A). In particular, the high-LHX2-expression group scored higher in both immune scores and estimated scores, which may be indicative of the high immune infiltration of BRCA (Figure 5B). In more detail, Th1 cells, Th2 cells, Treg, and activated DC (aDC) cells had a positive correlation with the expression of LHX2 in BRCA (Figure 5C,D). For a further immune infiltration analysis, we divided the 4T1 cells into the si-NC group and the si-LHX2 group (LHX2 knockdown). Subcutaneous tumor formation was then performed in C57BL/6 mice. Subsequently, the tumor tissues were analyzed using immunohistochemistry and immunofluorescence. We used immunohistochemistry to detect the different expressions of CD3 and CD4 in the tumor tissues of the two groups. CD3 marked tumor-infiltrating T cells, which play a vital role in the TME. We found that the CD3 level in the si-LHX2 group was significantly lower than that in the NC group (Figure 5E), which indicates that the expression of tumor-infiltrating T cells was positively correlated with the expression level of LHX2. CD4 marked CD4 + T cells, including Th2 cells and Th1 cells, which were positively correlated with LHX2 expression levels and the highest correlation coefficient in the bioinformatics analysis. The immunohistochemical results showed that the CD4 level of the si-LHX2 group was also lower than that of the NC group (Figure 5E), and the results were statistically significant, which was consistent with the results of the bioinformatics analysis. To further explore the effect of LHX2 on the expression levels of CD3 and CD4, as well as their relative expression levels, we performed immunofluorescence double-labeling experiments. The results were consistent with the immunohistochemistry results, and CD4/CD3 was also decreased in the si-LHX2 group (Figure 5F). In conclusion, the expression level of LHX2 is related to the immune infiltration of breast cancer.

### 3.6. LHX2 Regulates BRCA Cell Proliferation Migration, Invasion Ability, and Apoptosis In Vitro

**Figure 6:**
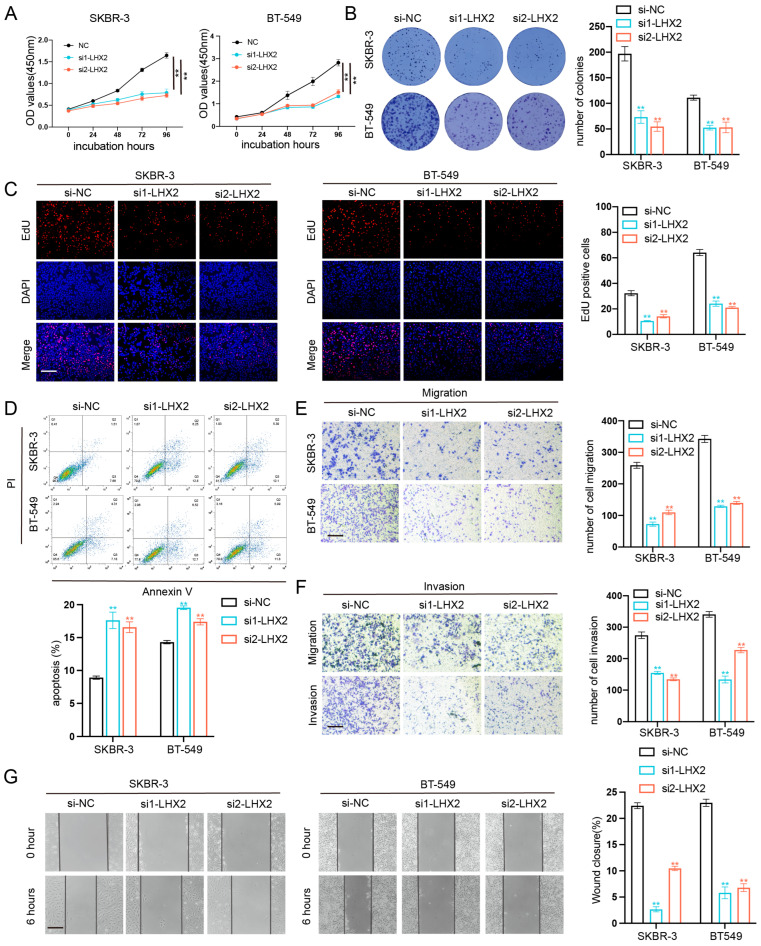
Downregulation of LHX2 inhibited breast cancer cell proliferation, migration, and invasion ability and promoted apoptosis in vitro. (**A**) CCK8 assays were performed to determine proliferation of SKBR-3 and BT-549 cells after transfection of siRNA of LHX2. ** *p* < 0.01. (**B**) Colony formation assays of SKBR-3 and BT-549 cells after downregulation of LHX2. ** *p* < 0.01. (**C**) EdU assays of SKBR-3 and BT-549 cells transfected with siRNA of LHX2. ** *p* < 0.01, scale bar 100 μM. (**D**) Apoptosis in SKBR-3 and BT-549 cells after transfection of shRNA of LHX2 analyzed using flow cytometry. ** *p* < 0.01. (**E**,**F**) Transwell assays were used to investigate the changes in migration and invasiveness capability of SKBR-3 and BT-549 cells after downregulation of LHX2. ** *p* < 0.01, scale bar 100 μM. (**G**) Wound healing assays were used to investigate the changes in migration capability of SKBR-3 and BT-549 cells after downregulation of LHX2. ** *p* < 0.01, scale bar 100 μM.

**Figure 7:**
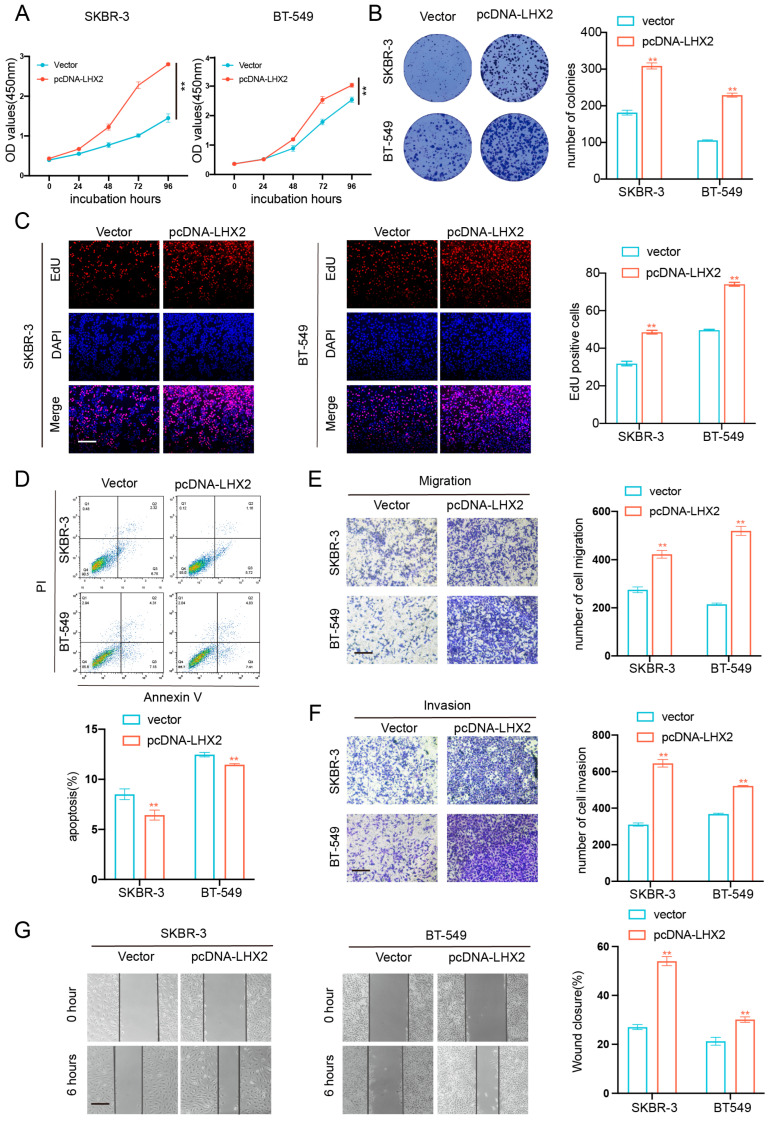
Overexpression of LHX2 promoted breast cancer cell proliferation, migration, and invasion ability and suppressed apoptosis in vitro. (**A**) CCK8 assays detected proliferation of SKBR-3 and BT-549 cells after overexpression of LHX2. ** *p* < 0.01. (**B**) Colony formation assays of SKBR-3 and BT-549 cells transfected with an overexpression plasmid of LHX2. ** *p* < 0.01. (**C**) EdU assays of SKBR-3 and BT-549 cells after transfection of overexpression plasmid of LHX2. ** *p* < 0.01, scale bar 100 μM. (**D**) Apoptosis in SKBR-3 and BT-549 cells after transfection of overexpression plasmid of LHX2 analyzed using flow cytometry. ** *p* < 0.01. (**E**,**F**) Transwell assays investigated the changes in migration and invasiveness capabilities of SKBR-3 and BT-549 cells after overexpression of LHX2. ** *p* < 0.01, scale bar 100 μM. (**G**) Wound healing assays investigated the changes in migration capability of SKBR-3 and BT-549 cells after overexpression of LHX2. ** *p* < 0.01, scale bar 100 μM.

To confirm the role of LHX2 in breast cancer, we detected the expression level of LHX2 in breast cancer cell lines, and the two cell lines with the highest LHX2 expression were selected for subsequent functional experiments (Figure S2A). Then, we manipulated the expression of LHX2 in SKBR-3 and BT-549 cells via siRNA-mediated downregulation and plasmid-mediated overexpression (Figure S2B,C). The CCK-8 assay showed that the proliferation of BRAC cells was inhibited upon the downregulation of LHX2 (Figure 6A) and that it was promoted when LHX2 was upregulated (Figure 7A). The clonogenic capacity was significantly decreased following the downregulation of LHX2 (Figure 6B). On the contrary, overexpressed LHX2 promoted the clonogenic capacity (Figure 7B). Consistently, we obtained the same results in the EdU assay (Figure 6C and Figure 7C). Then, the cell apoptosis assay indicated that the number of apoptosis cells increased in LHX2-silenced SKBR-3 and BT-549 cells (Figure 6D), while the apoptosis ability was restrained in LHX2-overexpressed cells (Figure 7D). Furthermore, Transwell assays revealed that the downregulation of LHX2 suppressed the cell migration and invasion of SKBR-3 and BT-549 cells, while the upregulation of LHX2 promoted them (Figure 6E,F and Figure 7E,F). The wound healing assays also verified that LHX2 enhanced the migration ability of SKBR-3 and BT-549 cells (Figure 6G and Figure 7G).

### 3.7. *LHX2* Regulates BRCA Cell Proliferation In Vivo

**Figure 8:**
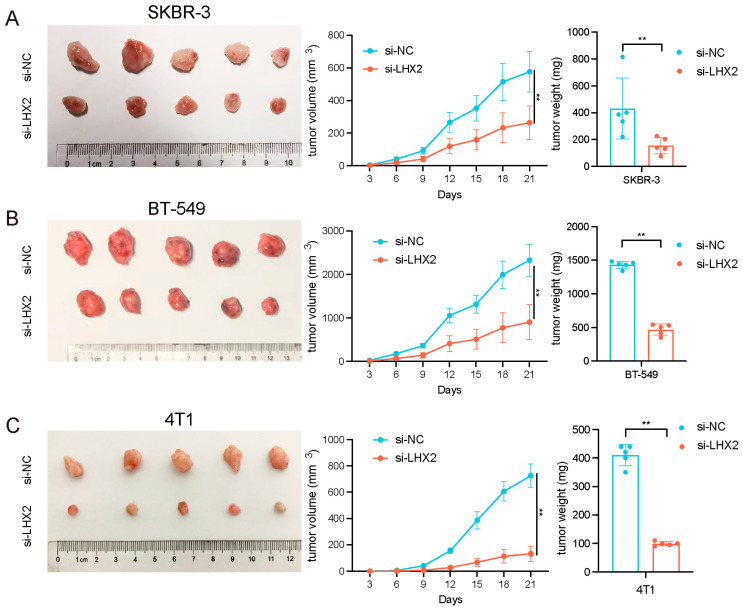
LHX2 regulates breast cancer cell proliferation in vivo. (**A**,**B**) shLHX2 was stably transfected into SKBR-3 and BT-549 cells and then injected into nude mice. (**C**) 4T1 cells were stably transfected with shLHX2 and then injected into C57BL/6 mice. Tumor volumes were calculated using the following formula: (shortest diameter)/2 × (longest diameter)/2. Tumor weights are represented as the means of tumor weights ± S.D. (standard deviation). ** *p* < 0.01.

To further validate whether LHX2 influenced the cell proliferation of breast cancer in vivo, SKBR-3 and BT-549 cells were stably transfected with sh-LHX2 and a control vector, and they were inoculated into BALB/c nude mice subcutaneously in the left and right axilla, respectively. Tumor volumes were calculated every 3 days after injection. Three weeks after inoculation, the weights of the tumors were recorded. The results show that the downregulation of LHX2 inhibited the cell proliferation of breast cancer in vivo (Figure 8A,B).

In addition, 4T1 cells, a murine cell line, were stably transfected with sh-LHX2 and a control vector. Then, the 4T1 cells from the si-NC and si-LHX2 groups were injected into the left and right axilla of C57BL/6 mice, respectively. The longest and shortest diameters of the tumor were measured every three days thereafter to calculate the tumor volume. After 21 days, the tumors were removed and weighed. We found that LHX2 also inhibited the cell proliferation of 4T1 cells (Figure 8C). Immunohistochemistry, immunofluorescence, and a TUNEL assay were performed on these tumor tissues to explore the mechanism of LHX2 in breast cancer immune infiltration and apoptosis.

### 3.8. *LHX2* Activates the PI3K/AKT/mTOR Pathway and Apoptosis Pathway in Breast Cancer

**Figure 9:**
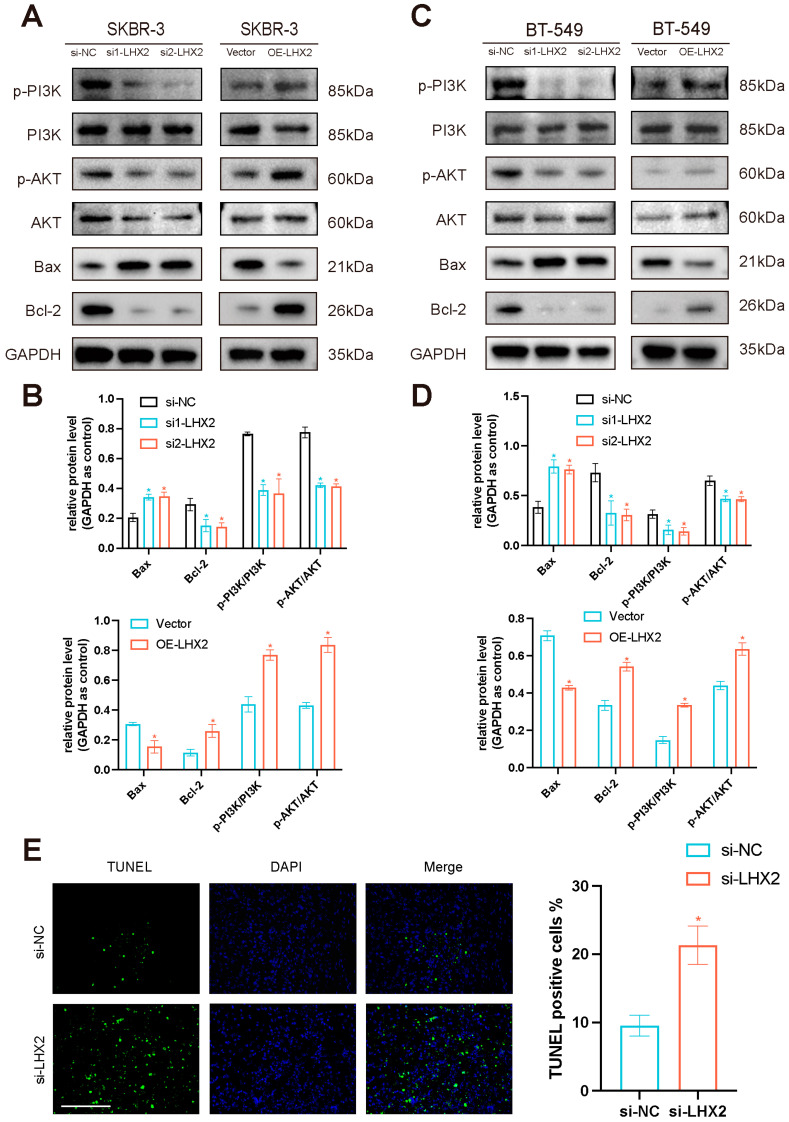
LHX2 regulates the PI3K/AKT/mTOR pathway and apoptosis pathway in breast cancer. (**A**) Western blot assays detected the expressions of Bax, Bcl-2, p-PI3K, PI3K, p-AKT, and AKT after downregulation and overexpression of LHX2 in SKBR-3 cells. (**B**) Statistics of relative protein expression levels in SKBR-3 cells. * *p* < 0.05. (**C**) Western blot assays detected the expressions of Bax, Bcl-2, p-PI3K, PI3K, p-AKT, and AKT after downregulation and overexpression of LHX2 in BT-549 cells. (**D**) Statistics of relative protein expression levels in BT-549 cells. * *p* < 0.05. (**E**) TUNEL assays of tumor tissues of 4T1 cells. * *p* < 0.05. The uncropped blots are shown in File S1.

According to our previous GSEA results, the PI3K/AKT/mTOR pathway and apoptosis pathway were enriched in the high-LHX2-expression phenotype. As was reported, LHX2 can promote the malignant behaviors of tumors through AKT signaling [11]. AKT can promote the progression of breast cancer through the action of HSP90 [12]. The PI3K/AKT pathway played a major role in tumorigenesis [13,14,15,16], and it interacts with the apoptosis pathway by inhibiting Bcl-2 [17]. LHX2 was found to induce autophagy through mTOR signaling [18]. To establish whether LHX2 activates the PI3K/AKT/mTOR pathway and apoptosis pathway in breast cancer, we conducted a Western blotting assay to detect the signature proteins in these pathways, including PI3K, p-PI3K, Akt, p-Akt, Bcl-2, and Bax. Phosphoinositide 3-kinase (PI3K) and Akt served to maintain cell proliferation and apoptosis [17]; P-Akt and p-PI3K are their active forms. Akt is a key effector of PI3K and an important survival kinase [19]; Akt signaling contributes to tumor growth and progression [20]. Bcl-2 inhibits apoptosis, and Bax is an important protein that promotes apoptosis; the functional interaction between them is conserved [21]. As a result, LHX2 overexpression upregulated p-PI3K, p-Akt, and Bcl-2, while it suppressed Bax in both SKBR-3 cells and BT-549 cells. By contrast, the LHX2 downregulation groups recorded the opposite results. The Western blot results indicated that LHX2 activated the PI3K/AKT/mTOR pathway and apoptosis pathway in breast cancer, and it inhibited the apoptosis of BRCA (Figure 9A–D). We also performed a TUNEL assay on tumor tissues, and compared with the si-NC group, the TUNEL-positive cells of si-LHX2 were significantly increased (Figure 9E), indicating that LHX2 inhibited apoptosis in breast cancer, which was consistent with the results of the Western blotting assay; thus, the results complemented each other.

## 4. Discussion

According to the cancer statistics for 2020, breast cancer accounts for about 30% of female cancers worldwide, which makes it a threat to the public’s health [1]. Breast cancer is a heterogeneous disease, whose molecular hallmarks have been comprehensively defined [22]. However, there is still an urgent need to identify new biomarkers to enrich the prognostic algorithms of breast cancer.

LHX2, a LIM homeobox gene, is a nuclear transcription co-factor that interacts with a myriad of transcriptional regulators and controls the expression of multiple genes and cellular functions [6]. Normally, LHX2 is necessary for the normal development of the eye, cerebral cortex, and efficient definitive erythropoiesis [4], and it plays a vital role in the maintenance of epithelial stem cells [23]. It is also decisive in producing neurons [24] and regulating early neural differentiation in humans [25]. Besides the critical physiologic role in embryonic development, the aberrant expression of LHX2 is associated with primary tumor growth and metastasis. For instance, the overexpression of LHX2 promotes the development of chronic myeloid leukemia [26], pancreatic ductal carcinoma [27], nasopharyngeal carcinoma [28], and non-small-cell lung cancer [29]. However, the downregulation of LHX2 correlated with intermediate, high-risk tumors and low patient survival in hepatoblastoma, which indicated that LHX2 plays complex roles in human cancers [6].

Previous studies showed that LHX2 promotes mammary carcinogenesis in mice [30]. Gao et al. discussed the diagnostic and prognostic value of LHXs in breast cancer [31]. Our study further discussed the prognostic value of LHX2 in breast cancer and its related mechanisms. Importantly, a lot of experiments were conducted to prove the results of the bioinformatics analysis.

As the role of LHX2 in breast cancer has yet to be thoroughly illustrated, we concentrated on the prognostic value and immune infiltration of LHX2 in our study. Given the favorable prognostic value and clinical relevance of LHX2, we then explored its function in BRCA. The major pathways enriched by GSEA were associated with metabolism and immunity; these have been found to be the most complex components in cancer research, and they always have interactions with each other. Research shows that abnormal metabolism is connected to immune dysfunction in cancer [32]. The tumor microenvironment plays a vital role in tumorigenesis, and it may provide multiple targets for cancer therapy [33]. Tumor-infiltrating lymphocytes consist of all lymphocytic cells that invade the tumor tissue and are a necessary part of the TME of most solid tumors, including BRCA [34]. There is increasing evidence for the clinical relevance of TILs in BRCA, particularly in HER2+ and TNBC subtypes [35]. Our analysis of immune infiltration demonstrated that the infiltration levels of Th2 cells, Th1 cells, Treg, and activated DC cells had a significant positive correlation with the expression of LHX2. Th2 cells, Th1 cells, and Treg are all categorized as CD4 + T helper cells, and they play an integral role in the tumor immune response [36,37]. Immunohistochemistry and immunofluorescence assays also confirmed that LHX2 promoted the expressions of infiltrating T cells and CD4 + T cells. Th2 cells always give rise to a humoral immune response that promotes tumor growth through the secretion of IL-10, IL-4, and TGF-β [34]; by contrast, Th1 cells can secrete IFN-γ, TNF-α, and IL-12 to mediate the anti-tumor response and provide successful protection against cancer [38]. The ongoing NCT03112590 trial suggests that boosting the Th1 immune response can achieve therapeutic benefits in breast cancer [34]. Th1 and Th2 cells have antagonistic functions, and an imbalance between them always results in decreased overall survival in BRCA [39]. The balance between Th1 and Th2 cells depends on the expression and activation of key molecules within the microenvironment of both activated naïve CD4+ T cells and the cell itself [36]. Tregs suppress anti-tumor immune responses via the secretion of IL-10 and the sequestration of IL-2 [40], and the infiltration of a large quantity of Tregs is often connected with a poor prognosis [37]. DC cells are fundamental for tumor-related antigen presentation to naïve T cells, resulting in tumor infiltration [41]. In conclusion, LHX2-related immune infiltration is quite complex, and its elucidation may require further studies.

Through in vitro and in vivo experiments, we found that LHX2 promoted cell proliferation, invasion ability, and migration in breast cancer, whereas it suppressed apoptosis in breast cancer. Then, we discovered that LHX2 activated the PI3K/AKT/mTOR pathway and the apoptosis pathway, enriched by GSEA in the high-LHX2-expression phenotype, by detecting signature proteins. The PI3K/AKT/mTOR pathway can regulate apoptosis [17] and has an influence on immune cell function and the modulation of the TME. For example, this pathway is necessary for maintaining the immunosuppressive function of Tregs [42]. Multiple studies have shown that metabolic modulation in immune cells is crucial for controlling anti-tumor immunity in the TME [39]. Overall, the PI3K/AKT/mTOR pathway is the most frequently mutated network in breast cancer and provides multiple molecular targets [43].

In the future, we may be able to carry out more experiments to study the deeper mechanism of LHX2’s regulation of breast cancer proliferation and migration and to also reveal how LHX2 is involved in the regulation of the tumor immune microenvironment of breast cancer.

## 5. Conclusions

In summary, LHX2 is highly expressed and has potential prognostic value in many malignancies, particularly in BRCA. The clinicopathological characteristics of BRCA have a close relationship with the expression of LHX2. Function enrichment analyses and GSEA allowed us to determine that LHX2-related genes centered on immunity and metabolism in BRCA; this was echoed by the immune infiltration related to LHX2 in BRCA. In vivo and in vitro experiments confirmed that LHX2 promoted the cell proliferation, migration ability, and invasion ability of BRCA but decreased apoptosis. A high expression of LHX2 in BRCA promoted the PI3K/AKT/mTOR pathway and apoptosis pathway. Encouragingly, our study found that LHX2 correlated with immune infiltration and metabolism, according to a functional enrichment analysis, GSEA, and an immune infiltration analysis. LHX2 was also highly expressed in HER-2-positive breast cancer and TNBC, which have more clinical benefits in immunotherapy than other subtypes of BRCA. It has been reported that immunity and metabolism have a close relationship with each other in tumorigenesis [39]. In conclusion, it is reasonable to assume that LHX2 can be a prognostic biomarker that is associated with immunity in BRCA. However, the tumor microenvironment is quite complex, and different kinds of TILs have a complicated influence on tumorigenesis, whether antagonistic or synergistic; therefore, much work is still needed to completely elucidate it.

## Data Availability

The data presented in this study are available in this article and Supplementary Materials.

## Supplementary Materials

The following supporting information can be downloaded at https://www.mdpi.com/article/10.3390/cancers15102773/s1, Figure S1: Prognostic analysis of LHX2 in different malignancies in TCGA database; Figure S2: Expression level of LHX2 and manipulation of the expression of LHX2 in breast cancer cells; Table S1: Sample size for bioinformatics analysis; Table S2: Data set; Table S3: Antibodies and primers; File S1: Full pictures of the Western blots.

## References

[1] Siegel R.L., Miller K.D., Jemal A. (2020). Cancer statistics, 2020. CA Cancer J. Clin..

[2] Harbeck N., Gnant M. (2017). Breast cancer. Lancet.

[3] Liang Y., Zhang H., Song X., Yang Q. (2020). Metastatic heterogeneity of breast cancer: Molecular mechanism and potential therapeutic targets. Semin. Cancer Biol..

[4] Porter F.D., Drago J., Xu Y., Cheema S.S., Wassif C., Huang S.P., Lee E., Grinberg A., Massalas J.S., Bodine D. (1997). Lhx2, a LIM homeobox gene, is required for eye, forebrain, and definitive erythrocyte development. Development.

[5] Li X., Wu X., Chen H., Liu Z., He H., Wang L. (2022). LHX2 Enhances the Malignant Phenotype of Esophageal Squamous Cell Carcinoma by Upregulating the Expression of SERPINE2. Genes.

[6] Mosca N., Khoubai F.Z., Fedou S., Carrillo-Reixach J., Caruso S., Del Rio-Alvarez A., Dubois E., Avignon C., Dugot-Senant N., Guettier C. (2022). LIM Homeobox-2 Suppresses Hallmarks of Adult and Pediatric Liver Cancers by Inactivating MAPK/ERK and Wnt/Beta-Catenin Pathways. Liver Cancer.

[7] Emens L.A. (2018). Breast Cancer Immunotherapy: Facts and Hopes. Clin. Cancer Res..

[8] Subramanian L., Sarkar A., Shetty A.S., Muralidharan B., Padmanabhan H., Piper M., Monuki E.S., Bach I., Gronostajski R.M., Richards L.J. (2011). Transcription factor Lhx2 is necessary and sufficient to suppress astrogliogenesis and promote neurogenesis in the developing hippocampus. Proc. Natl. Acad. Sci. USA.

[9] Gao W., Wang X., Zhou Y., Wang X., Yu Y. (2022). Autophagy, ferroptosis, pyroptosis, and necroptosis in tumor immunotherapy. Signal Transduct. Target. Ther..

[10] Dieci M.V., Miglietta F., Guarneri V. (2021). Immune Infiltrates in Breast Cancer: Recent Updates and Clinical Implications. Cells.

[11] Liu J., Li X., Yang S., Mou J., Lu H. (2020). Whole exome sequencing and transcriptome-wide profiling identify potentially subtype-relevant genes of nasopharyngeal carcinoma. Pathol. Res. Pract..

[12] Alberti G., Vergilio G., Paladino L., Barone R., Cappello F., de Macario E.C., Macario A.J.L., Bucchieri F., Rappa F. (2022). The Chaperone System in Breast Cancer: Roles and Therapeutic Prospects of the Molecular Chaperones Hsp27, Hsp60, Hsp70, and Hsp90. Int. J. Mol. Sci..

[13] Hoxhaj G., Manning B.D. (2020). The PI3K-AKT network at the interface of oncogenic signalling and cancer metabolism. Nat. Rev. Cancer.

[14] Miricescu D., Totan A., Stanescu S., Badoiu S.C., Stefani C., Greabu M. (2020). PI3K/AKT/mTOR Signaling Pathway in Breast Cancer: From Molecular Landscape to Clinical Aspects. Int. J. Mol. Sci..

[15] Shorning B.Y., Dass M.S., Smalley M.J., Pearson H.B. (2020). The PI3K-AKT-mTOR Pathway and Prostate Cancer: At the Crossroads of AR, MAPK, and WNT Signaling. Int. J. Mol. Sci..

[16] Xue C., Li G., Lu J., Li L. (2021). Crosstalk between circRNAs and the PI3K/AKT signaling pathway in cancer progression. Signal Transduct. Target. Ther..

[17] Fruman D.A., Chiu H., Hopkins B.D., Bagrodia S., Cantley L.C., Abraham R.T. (2017). The PI3K Pathway in Human Disease. Cell.

[18] Song H., Liu J., Wu X., Zhou Y., Chen X., Chen J., Deng K., Mao C., Huang S., Liu Z. (2019). LHX2 promotes malignancy and inhibits autophagy via mTOR in osteosarcoma and is negatively regulated by miR-129-5p. Aging.

[19] Yang Q., Guan K.-L. (2007). Expanding mTOR signaling. Cell Res..

[20] Manning B.D., Toker A. (2017). AKT/PKB Signaling: Navigating the Network. Cell.

[21] Gaumer S., Guénal I., Brun S., Théodore L., Mignotte B. (2000). Bcl-2 and Bax mammalian regulators of apoptosis are functional in Drosophila. Cell Death Differ..

[22] Loibl S., Poortmans P., Morrow M., Denkert C., Curigliano G. (2021). Breast cancer. Lancet.

[23] Tiede S., Paus R. (2006). Lhx2—Decisive role in epithelial stem cell maintenance, or just the “tip of the iceberg”?. Bioessays.

[24] Muralidharan B., Khatri Z., Maheshwari U., Gupta R., Roy B., Pradhan S.J., Karmodiya K., Padmanabhan H., Shetty A.S., Balaji C. (2017). LHX2 Interacts with the NuRD Complex and Regulates Cortical Neuron Subtype Determinants Fezf2 and Sox11. J. Neurosci..

[25] Hou P.-S., Chuang C.-Y., Kao C.-F., Chou S.-J., Stone L., Ho H.-N., Chien C.-L., Kuo H.-C. (2013). LHX2 regulates the neural differentiation of human embryonic stem cells via transcriptional modulation of PAX6 and CER1. Nucleic Acids Res..

[26] Wu H.K., Minden M.D. (1997). Transcriptional activation of human LIM-HOX gene, hLH-2, in chronic myelogenous leukemia is due to a cis-acting effect of Bcr-Abl. Biochem. Biophys. Res. Commun..

[27] Zhou F., Gou S., Xiong J., Wu H., Wang C., Liu T. (2014). Oncogenicity of LHX2 in pancreatic ductal adenocarcinoma. Mol. Biol. Rep..

[28] Xie T., Du K., Liu W., Liu C., Wang B., Tian Y., Li R., Huang X., Lin J., Jian H. (2022). LHX2 facilitates the progression of nasopharyngeal carcinoma via activation of the FGF1/FGFR axis. Br. J. Cancer.

[29] Shi X., Zhan L., Xiao C., Lei Z., Yang H., Wang L., Zhao J., Zhang H.-T. (2015). miR-1238 inhibits cell proliferation by targeting LHX2 in non-small cell lung cancer. Oncotarget.

[30] Kuzmanov A., Hopfer U., Marti P., Meyer-Schaller N., Yilmaz M., Christofori G. (2014). LIM-homeobox gene 2 promotes tumor growth and metastasis by inducing autocrine and paracrine PDGF-B signaling. Mol. Oncol..

[31] Gao P., Sun N., Zhao T., Sun Y., Gu J., Ma D., Tian H., Peng Z., Zhang Y., Han F. (2022). Identification of prognostic indicators, diagnostic markers, and possible therapeutic targets among LIM homeobox transcription factors in breast cancer. Cancer Innov..

[32] Stienstra R., Netea-Maier R.T., Riksen N.P., Joosten L.A., Netea M.G. (2017). Specific and Complex Reprogramming of Cellular Metabolism in Myeloid Cells during Innate Immune Responses. Cell Metab..

[33] Xiao Y., Yu D. (2021). Tumor microenvironment as a therapeutic target in cancer. Pharmacol. Ther..

[34] Basu A., Ramamoorthi G., Jia Y., Faughn J., Wiener D., Awshah S., Kodumudi K., Czerniecki B.J. (2019). Immunotherapy in breast cancer: Current status and future directions. Adv. Cancer Res..

[35] Dieci M.V., Radosevic-Robin N., Fineberg S., van den Eynden G., Ternes N., Penault-Llorca F., Pruneri G., D’alfonso T.M., Demaria S., Castaneda C. (2018). International Immuno-Oncology Biomarker Working Group on Breast, C., Update on tumor-infiltrating lymphocytes (TILs) in breast cancer, including recommendations to assess TILs in residual disease after neoadjuvant therapy and in carcinoma in situ: A report of the International Immuno-Oncology Biomarker Working Group on Breast Cancer. Semin. Cancer Biol..

[36] Sanders V.M. (2006). Epigenetic regulation of Th1 and Th2 cell development. Brain Behav. Immun..

[37] Tanaka A., Sakaguchi S. (2017). Regulatory T cells in cancer immunotherapy. Cell Res..

[38] Datta J., Fracol M., McMillan M.T., Berk E., Xu S., Goodman N., Lewis D.A., DeMichele A., Czerniecki B.J. (2016). Association of Depressed Anti-HER2 T-Helper Type 1 Response with Recurrence in Patients with Completely Treated HER2-Positive Breast Cancer: Role for Immune Monitoring. JAMA Oncol..

[39] Speiser D.E., Ho P.C., Verdeil G. (2016). Regulatory circuits of T cell function in cancer. Nat. Rev. Immunol..

[40] Zou W. (2006). Regulatory T cells, tumour immunity and immunotherapy. Nat. Rev. Immunol..

[41] Steinman R.M. (2012). Decisions about dendritic cells: Past, present, and future. Annu. Rev. Immunol..

[42] O’Donnell J.S., Massi D., Teng M.W.L., Mandala M. (2018). PI3K-AKT-mTOR inhibition in cancer immunotherapy, redux. Semin. Cancer Biol..

[43] Guerrero-Zotano A., Mayer I.A., Arteaga C.L. (2016). PI3K/AKT/mTOR: Role in breast cancer progression, drug resistance, and treatment. Cancer Metastasis Rev..

