# Supplementary material for: LHX2 Is a Potential Biomarker and Associated with Immune Infiltration in Breast Cancer
Source: Cancers (Basel). 2023 May 16;15(10):2773. doi: 10.3390/cancers15102773 (PMC10216828; doi:10.3390/cancers15102773)
Supplement: Supplementary file 1 [file cancers-15-02773-s001.zip › Figure supplement.pdf]

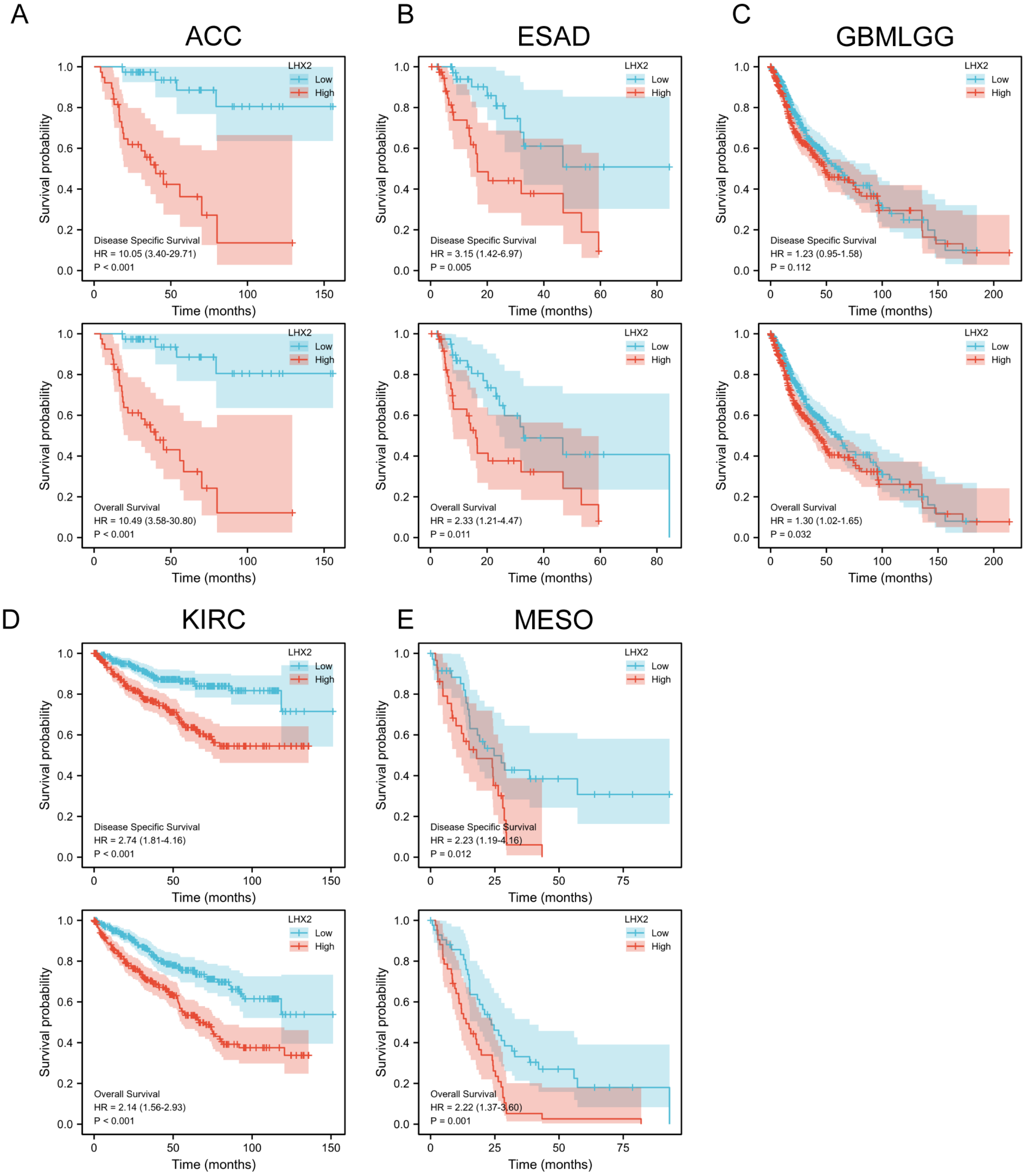

Figure S1. Prognostic analysis of LHX2 in different malignancies. **(A–E)** Kaplan–Meier overall survival (OS) and disease specific survival (DSS) for samples in high– and low–LHX2 groups in ACC **(A)**, ESAD **(B)**, GBMLGG **(C)**, KIRC **(D)** and MESO **(E)** in TCGA database.

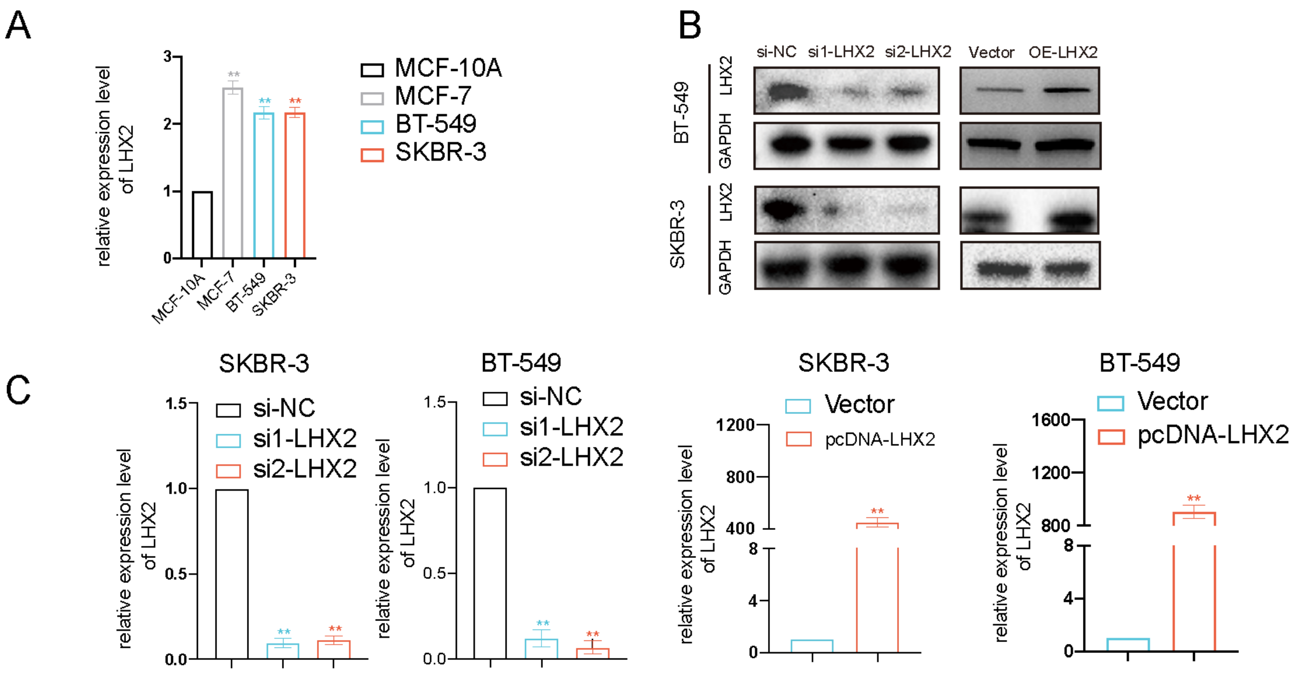

Figure S2 Expression level of LHX2 and manipulation of the expression of LHX2 in breast cancer cells. **(A)** Analysis of LHX2 expression levels in breast cancer cell lines and normal mammary epithelial cells by qRT–PCR. **(B)** Western–blot analysis of LHX2 expression level in SKBR–3 and BT549 cells after being transfected with siRNAs of LHX2 or overexpression plasmid of LHX2 **(C)** Analysis of LHX2 RNA expression level following treatment SKBR–3 and BT549 cells with siRNAs of LHX2 or overexpression plasmid of LHX2.
